# Embryonic exposure to the widely-used herbicide atrazine disrupts meiosis and normal follicle formation in female mice

**DOI:** 10.1038/s41598-017-03738-1

**Published:** 2017-06-14

**Authors:** Aurore Gely-Pernot, Souhila Saci, Pierre-Yves Kernanec, Chunxiang Hao, Frank Giton, Christine Kervarrec, Sergei Tevosian, Severine Mazaud-Guittot, Fatima Smagulova

**Affiliations:** 1 Inserm U1085 IRSET, 9 Avenue du prof Léon-Bernard, 35000 Rennes, France; 2 EHESP, 2 Avenue du Prof Léon-Bernard, 35000 Rennes, France; 3 Sorbonne University, 100-104 Avenue de France, 75013 Paris, France; 4 APHP CIB Henri Mondor; Inserm U955 IMRB, Team 7, Translational Research in Genito-Urinary Oncogenesis, Faculty of Medicine, 94000 Créteil, France; 5 University of Florida, Department of Physiological Sciences, Box 100144, 1333 Center Drive, 32610 Gainesville, FL USA

## Abstract

The widely-used herbicide atrazine (ATZ) is detected in ground and surface water in many countries. Several studies in animals have demonstrated that ATZ has endocrine-disrupting effects on male and female reproduction in many vertebrate species. In this study, we investigated the effects of ATZ exposure on meiosis, a key step in gametogenesis in mammals. The treatment was initiated before oocyte entry into meiosis, which occurs during the embryonic period in females. We found that embryonic exposure to ATZ increases the level of 8-oxo-guanine in the nucleus of meiotic cells, reflecting oxidative stress and affecting meiotic double-strand break repair, chromosome synapsis and crossover numbers. Finally, embryonic exposure to ATZ reduces the number of primordial follicles and increases the incidence of multi-oocyte follicles in adult mice. Our data demonstrate that embryonic exposure to ATZ disrupts prophase I of meiosis and affects normal follicle formation in female mice.

## Introduction

Pesticides and herbicides are widely used in agriculture in many countries, and their negative impacts on human health and wildlife are still not well understood. According to the World Health Organization report in 2012, pesticides negatively impact the reproductive system of humans and wildlife around the world^1–5^. Atrazine (ATZ), a widely-used herbicide, is detected in the groundwater of many countries, including the USA, Spain and Canada^6–8^. ATZ is still detected in countries where it has been banned^9–11^. The Natural Resources Defense Council (USA) reported that 40 tested watersheds had detectable levels of ATZ, with average levels above 1 ppb, while in some states (Indiana, Missouri, and Nebraska), ATZ exceeded 100 ppb in watersheds.

Low levels of ATZ metabolites in pregnant women are associated with low birth weight in newborns^12, 13^, suggesting possible negative ATZ-related effects on the early developmental program. This fact raises a serious concern of ATZ safety for humans.

In a series of studies in rodents, it has been shown that exposure to ATZ alters reproductive function in males and females^14–18^. Specifically, chronic exposure to ATZ during adulthood impairs folliculogenesis in female rats and leads to increased follicle atresia and the production of multi-oocyte follicles (MOF)^19^. The changes in follicular morphology were associated with abnormal endocrine function. It is suggested that the effects of ATZ on ovarian function in rats are mediated through Luteinizing Hormone (LH) and prolactin (PRL), which are known to control the secretion of steroid hormones^20^. It was also proposed that ATZ affects hypothalamic estrogen receptor function^21^. High doses of ATZ inhibit the LH surge and reduce the number of ovulated follicles in rats^22^. Exposure to ATZ significantly increases the reproductive cycle length in rats^23^. Exposure to ATZ in other animal species similarly has endocrine-disrupting effects. For example, ATZ reduces the circulating concentrations of 17β-estradiol (E2), progesterone (P4) and LH and increases PRL in quails^24^. In contrast, female pigs exposed to a low dose of ATZ exhibit a significant increase in serum E2 concentration^25^. The hormonal changes in exposed pigs are associated with morphological effects, including the appearance of multiple ovarian follicular cysts^25^. Taken together, these studies show ATZ-related effects on ovarian morphology and endocrine function in animals exposed at the adult stage. The effects of ATZ on early oocyte development and oocyte maturation are largely unknown. It has been suggested that exposure to environmental factors during early development can profoundly influence long-term health^26^. The early developmental stages in germ cells are characterized by global changes in epigenetic marks, chromatin remodeling events and resetting the gene network program due to somatic to germline transition. Primordial germ cells (PGCs), the first germline cell population, settle the developing urogenital ridges at embryonic day 11.5 (E11.5) in mice and initiate differentiation either toward a spermatogenic (male) or an oogenic (female) pathway. In females, oocytes proliferate until E12.5–13.5, when they start entering prophase I of meiotic division. In the fetal ovary, the homologous recombination (HR) of prophase I occurs between E13.5 and E18.5. HR is initiated by the formation of double-stand breaks (DSBs) throughout the entire genome. DSB formation is necessary for the homologous chromosome search. Failure of proper segregation following HR is associated with genomic rearrangements, mutations and aneuploidies^27, 28^. Chromosome segregation is mediated by the formation of the synaptonemal complex (SC). The SC is formed by axial/lateral elements along the chromosomal axis of each homologue containing synaptonemal complex protein SYCP3 and by a central connecting element containing SYCP1 protein^29^. Depending on the level of segregation of chromosomes, several substages of prophase I have been defined, including the *leptotene, zygotene, pachytene, diplotene* and *diakinesis* stages in mammals^29^. The DSBs are introduced at the late *leptotene* stage, and most of them are repaired before the *pachytene* stage via a so-called non-crossover pathway. One to four breaks per chromosome in mice could be repaired via the crossover pathway in which the parental chromosomes exchange the chromosome arms. The formation of crossovers is especially crucial as they are the only places where the homologous chromosomes are physically connected before the first meiotic division. The failure to produce at least one crossover per chromosome is associated with aneuploidy and nondisjunction. Just before birth, the oocytes undergo meiotic prophase I arrest, and homologous chromosomes remain physically connected at crossover locations until the oocytes reinitiate meiosis at puberty. The completion of the first meiotic division requires hormonal stimulation, while the second meiotic division is completed after fertilization. Both divisions end with the extrusion of a polar body.

Meiosis is particularly vulnerable to the influence of endogenous and exogenous factors and is more susceptible to aneuploidy^30^. We hypothesize that embryonic exposure to ATZ would impact prophase I of meiosis and impose changes that could affect the oocyte developmental program.

Here, we show that embryonic exposure to ATZ causes oxidative stress in oocytes, affects female meiosis, impairs DSB repair, delays the synapsis between homologous chromosomes and reduces crossover formation. Thereafter, embryonic exposure to ATZ reduces the number of primordial follicles and increases the incidence of MOF without notable changes in hormone concentrations. Exposure to ATZ leads to increases in 8-oxo-guanine (8-oxo-G) production in the nucleus. In summary, our data demonstrate immediate deleterious effects of ATZ exposure on female meiosis and delayed effects on ovarian differentiation.

## Results

### The dose of ATZ used in this study

We performed preliminary experiments using several doses of ATZ (0, 100, 150, and 300 mg/kg/day) in order to choose a single dose of treatment for a full analysis of ATZ-related impacts on the development of the mouse ovary. We found that mice poorly tolerate doses higher than 100 mg/kg/day. The 100 mg/kg/day dose is commonly used in many *in vivo* studies, including those using mice^31, 32^, rats^33^ and rabbits^34^. We thus chose this dose for the extended analysis of ATZ-related effects on the ovary.

### ATZ exposure does not alter the number of germ cells in embryonic ovaries

To analyze the effects of ATZ exposure on germ cell numbers we prepared sections of E18.5 control or treated ovaries and stained them with hematoxylin and eosin (H&E). The morphology of E18.5 H&E-stained ovaries from ATZ-exposed mice was similar to that of control females (Suppl. Fig. 1A,B). To evaluate whether the exposure to ATZ impacts germ cell numbers, E18.5 ovarian sections were immunostained against DDX4 for the quantification of total oocyte numbers (Suppl. Fig. 1C,D). ATZ exposure did not significantly affect germ cell numbers in E18.5 ATZ-exposed ovaries compared to controls (Suppl. Fig. 1E).

### Exposure to ATZ causes an increase in 8-oxo-G

**Figure 1:**
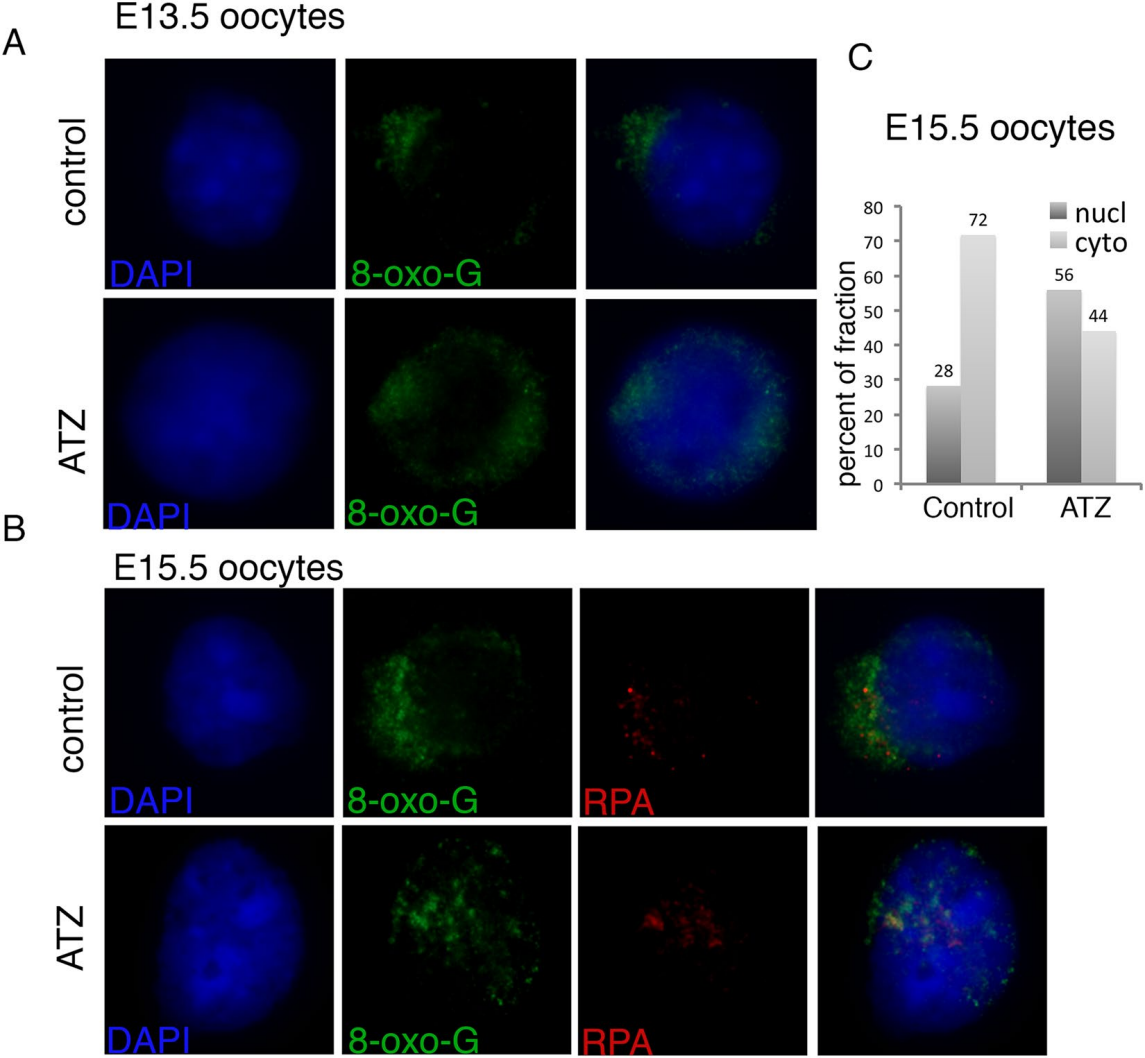
Embryonic exposure to ATZ increases the nuclear accumulation of 8-oxo-G. (**A**,**B**) Representative images of (**A**) E13.5 and (**B**) E15.5 oocytes immunostained with antibodies against 8-oxo-G (green), or RPA (red) are shown. DAPI staining is in blue. (**C**) Quantitative analysis of 8-oxo-G staining in E15.5 samples. At least 90 cells for each condition were analysed and data were expressed as % of cells with preferentially nuclear (nucl) or preferential cytoplasmic (cyto) staining.

Next, to reveal whether meiotic DSB repair is affected by increased production of 8-oxo-G, the most common DNA lesion produced in DNA, we performed immunostaining for 8-oxo-G on slides of E13.5 and E15.5 embryonic ovaries (Fig. 1). In E13.5 oocytes, 8-oxo-G staining was mainly detected in the cytoplasm in both the control and ATZ-treated samples (Fig. 1A). In E15.5 control oocytes, 8-oxo-G was mainly detected in the cytoplasm, with a detectable signal in the nucleus of only a small proportion of cells (Fig. 1B). In contrast, in oocytes derived from ATZ samples, we found an increased proportion of 8-oxo-G-positive nuclei. Since it has been suggested that replication protein A (RPA) is essential for bypassing the 8-oxo-G sites^35, 36^, we co-immunostained for RPA and 8-oxo-G. RPA was increased in the nuclei of ATZ treated oocytes at sites containing 8-oxo-G (Fig. 1B). The quantitative analysis of 8-oxo-G staining in E15.5 oocytes showed that the proportion of oocytes with nuclear staining increased by 2-fold in ATZ-exposed ovaries (Fig. 1C). Our data suggest that exposure to ATZ leads to oxidative stress in oocytes.

### The critical step of meiosis is affected in ATZ-exposed oocytes

**Figure 2:**
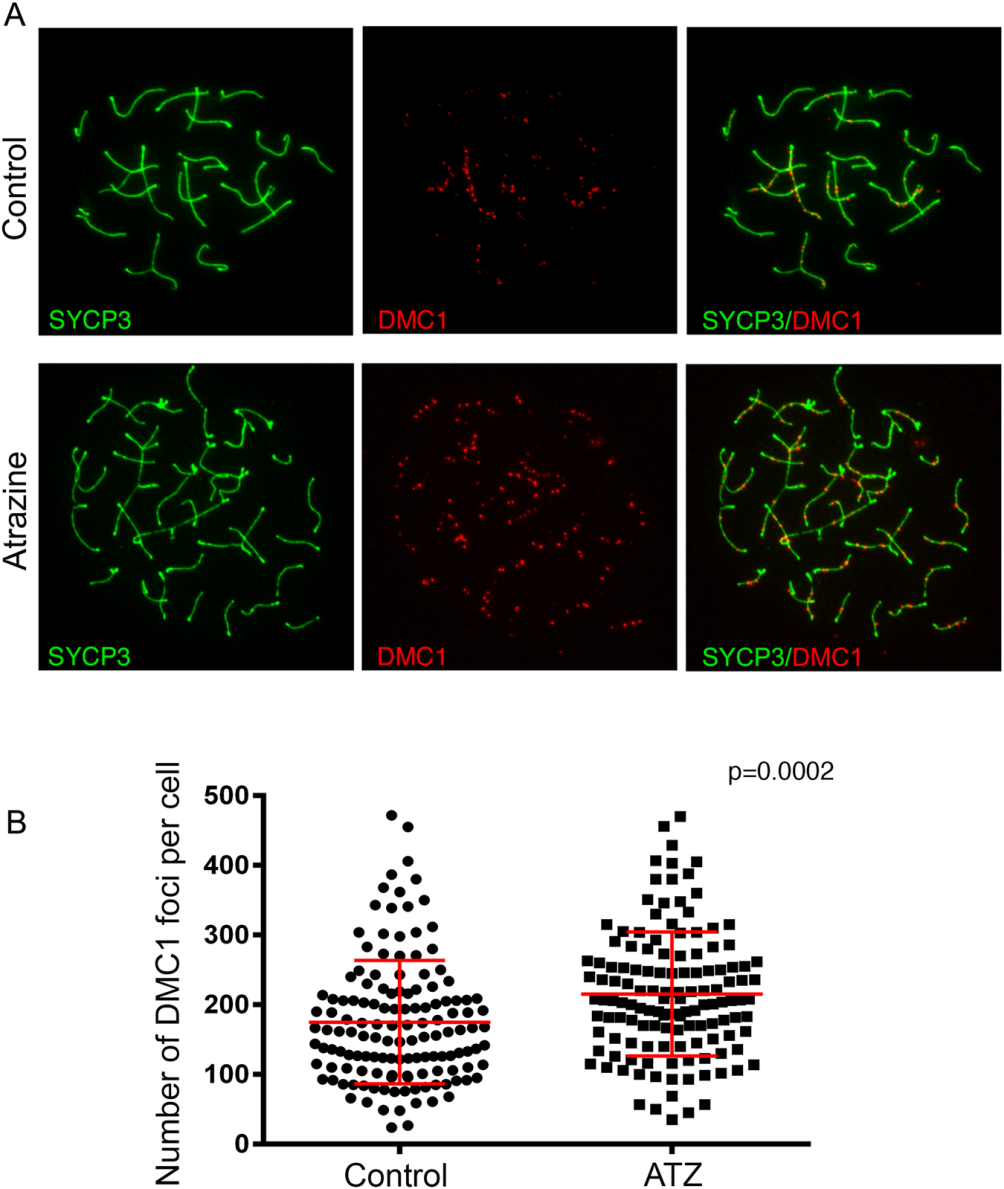
The number of DSBs per oocyte in E15.5 ATZ-exposed ovaries is significantly increased compared to controls. (**A**) Surface spreads from E15.5 control (top row) and ATZ-treated (bottom row) ovaries were immunostained with anti-DMC1 (red) and anti-SYCP3 (green) antibodies. In ATZ-exposed ovaries, the number of DMC1 foci was increased compared to controls. (**B**) Quantitative analysis of the number of DMC1 foci per cell performed on at least 100 oocytes,  oocytes were analyzed from four different animals for each condition (p = 0.0002, t-test). DMC1 and foci data are presented in plots as the mean values of DMC1 foci per cell +/−SD.

To analyze the effect of ATZ exposure on the early meiotic program, chromosomal spreads from E15.5 ovaries were immunostained for DMC1, the DNA-binding recombination protein that has a strong affinity for the single-stranded DNA tails of DSBs and for SYCP3, a component of the chromosome axis, to visualize the chromosomes (Fig. 2A). The mean number of DMC1 foci per oocyte was significantly higher in ATZ-treated oocytes (215 +/− 88.6; n = 134 oocytes) than in control oocytes (175 +/− 88.3, n = 138 oocytes; p = 0.0002, t-test), suggesting that exposure to ATZ affects the DNA repair process (Fig. 2B), as DSBs are not repaired efficiently in ATZ-treated oocytes.

### Homologous chromosome synapsis is incomplete in a fraction of ATZ-treated oocytes

**Figure 3:**
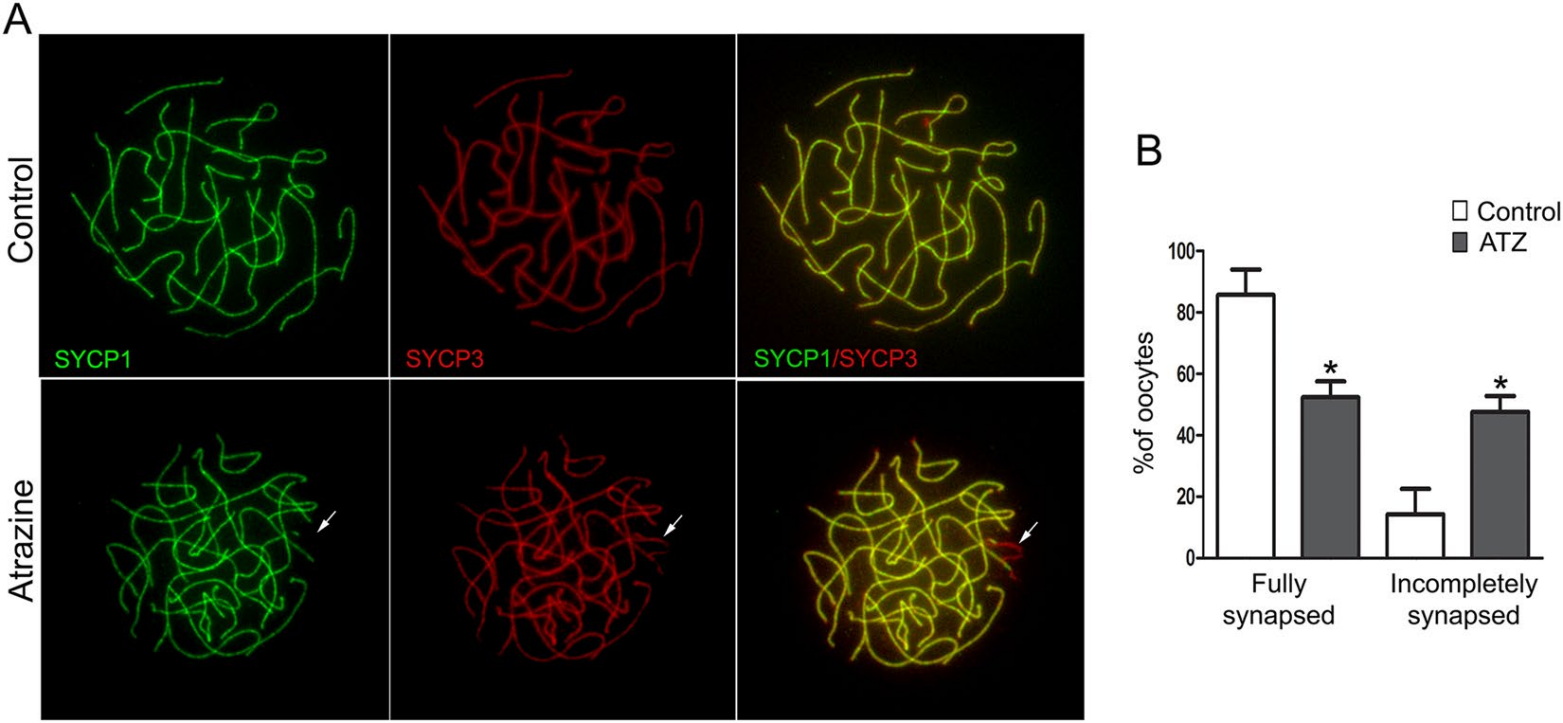
ATZ-exposed oocytes have increased numbers of chromosomes with incomplete synapsis. (**A**) Surface spreads from E16.5 control (top row) and ATZ-treated ovaries (bottom row) were stained with anti-SYCP1 (green) and anti-SYCP3 (red) antibodies. Note that in the control, the chromosome synapsis is completed, and the staining pattern of the synaptonemal proteins SYCP1 and SYCP3 are completely overlapped. In contrast, in ATZ-treated ovaries, the synapsis is not fully completed. The arrow indicates the part of the chromosomes with incomplete synapsis. (**B**) Quantification of the percentage of cells containing fully and partially synapsed chromosomes in at least 100 oocytes (*p ≤ 0.05, nonparametric Mann-Whitney test; n = 4). The data are presented as median values of fully and partially synapsed chromosomes +/−SD.

To further investigate whether ATZ-induced altered DSB repair subsequently affects the efficiency of meiotic synapsis, we performed a quantitative analysis of chromosome synapsis using E16.5 ATZ-treated and control ovarian spreads. We immunostained the spreads for SYCP1 protein, a component of the central element that appears only at fully synapsed regions of chromosomes, and for SYCP3. Cells were divided into two groups, totally or partially synapsed chromosomes, and both groups were quantified (Fig. 3A). Although a number of chromosomes in ATZ-treated oocytes appeared properly synapsed, many cells displayed incomplete synapsis (47.58 ± 10.33%, n = 119 oocytes) compared to controls (14.27 ± 16.52%; n = 140 oocytes; p < 0.05; nonparametric test, Mann-Whitney test) (Fig. 3A,B). These data suggest that chromosomes with incomplete meiotic synapsis are overrepresented (about 3 fold) in ATZ-exposed oocytes (Fig. 3B).

### Crossover formation is affected in ATZ-exposed oocytes

**Figure 4:**
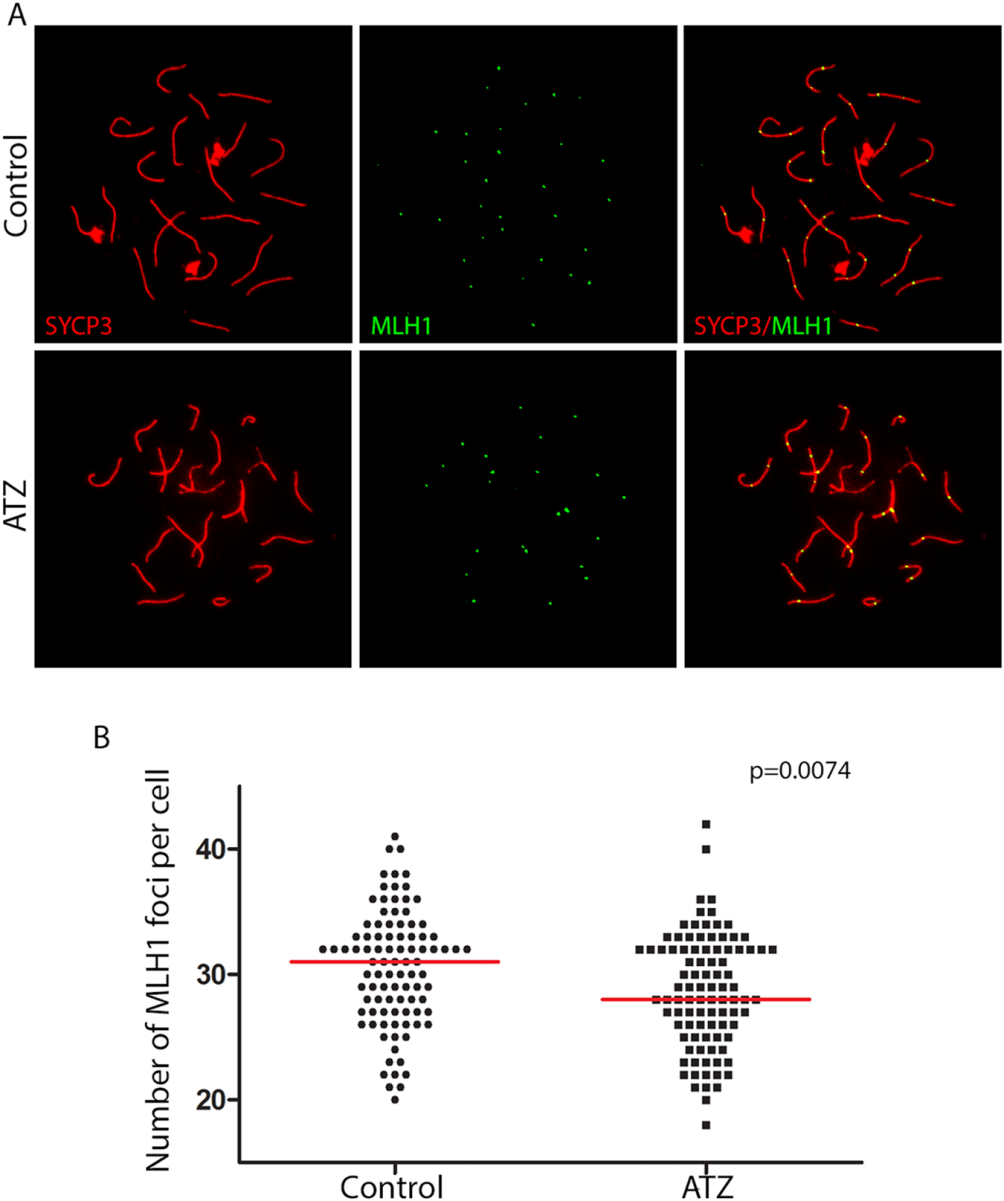
The formation of crossovers is decreased in ATZ-exposed ovaries. (**A**) Surface spreads from E18.5 control (top row) and ATZ-treated ovaries (bottom row) were immunostained with anti-SYCP3 (red) and anti-MLH1 (green) antibodies. (**B**) Quantification of the number of MLH1 foci per cell in control or ATZ-treated ovaries in at least 100 oocytes (oocytes were analyzed from four different animals for each condition; p = 0.0074, t-test). MLH1 foci data are presented in plots as the mean values of MLH1 foci per cell.

To examine how ATZ impacts meiotic crossover formation, we immunostained E18.5 ovarian chromosome spreads with antibodies against MutS homolog protein (MLH1), which appears at the sites of crossover formation at the *pachyetene* stage of prophase I (Fig. 4A). The quantitative analysis of MLH1 foci revealed that the mean value of crossover numbers per oocyte was significantly decreased in ATZ-exposed cells (28.8 ± 4.5, n = 95 oocytes) compared to control (30.5 ± 4.6; n = 95 oocytes) cells (Fig. 4B). These data suggest that exposure to ATZ leads to changes in crossover numbers when compared to control oocytes.

### The initial follicular wave is affected in ATZ-exposed animals

**Figure 5:**
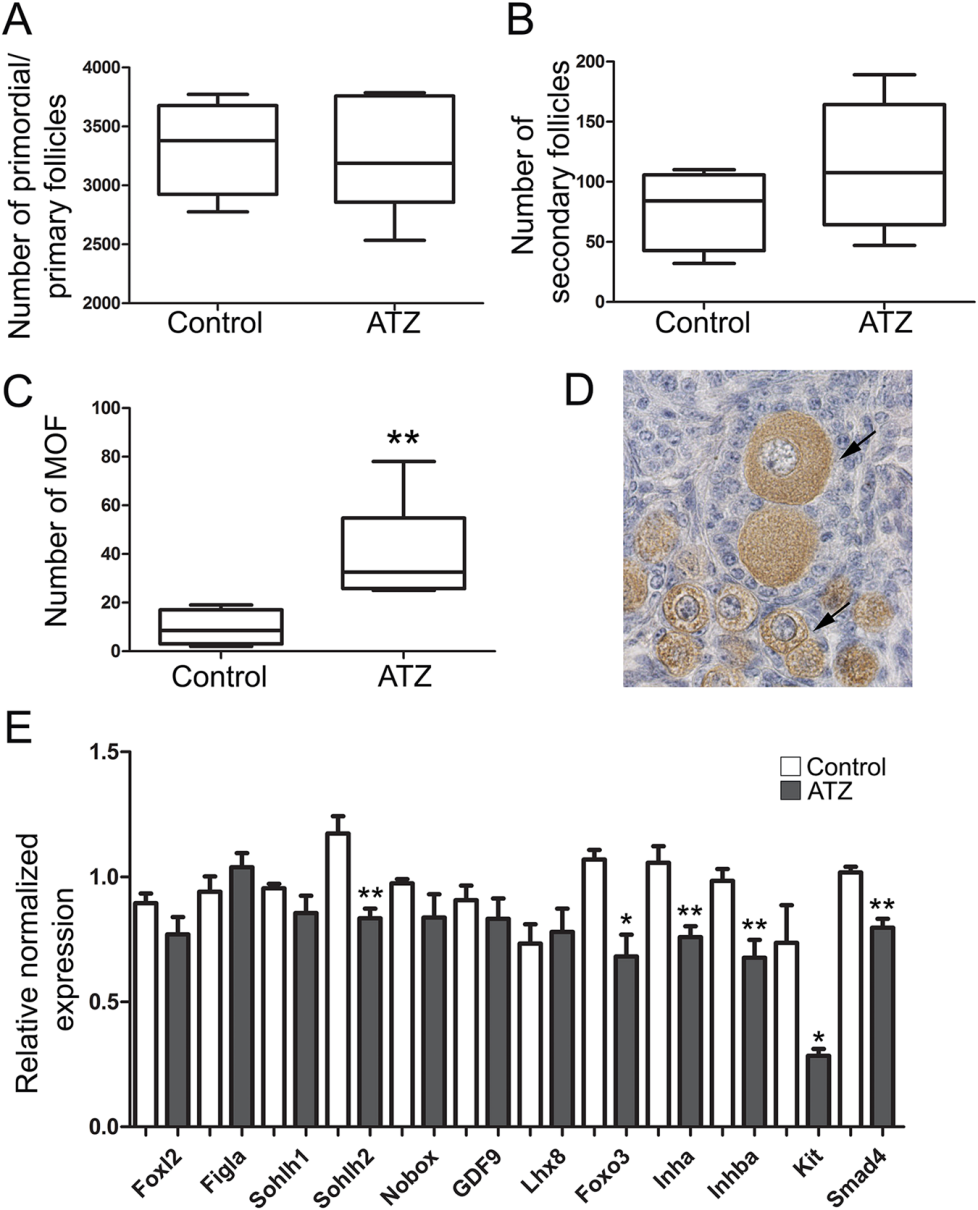
Exposure to Atrazine affects the initial follicular wave in mice. (**A**–**C**) Quantification of the number of (**A**) primordial and primary follicles, (**B**) secondary follicles and (**C**) multi-oocyte follicles (MOF) in 6-d.o. ovaries of control or ATZ-treated mice (control, n = 4; ATZ, n = 6; *p ≤ 0.05 and **p ≤ 0.01, nonparametric Mann-Whitney test). The data are presented as median values, and the lower and upper quartile values are shown. (**D**) A representative image of a paraffin section of an ATZ-treated ovary that was immunostained by MSY2, a specific marker of oocytes. The arrows indicate MOFs. (**E**) The expression levels of genes involved in follicular growth were analyzed by RT-qPCR using RNA from 6-d.o. ovaries from control and ATZ-treated animals (n = 5; and **p < 0.01, nonparametric Mann-Whitney test). The qPCR data are presented as the mean value +/−SEM.

To examine how in utero exposure to ATZ affects the oocyte pool, we counted the number of primordial follicles in 6-day-old (d.o.) control and ATZ-exposed ovaries on sections immunostained for the MSY2 protein, a marker of the oocyte^37^. The numbers of primordial, primary and secondary follicles were not altered in ATZ-exposed ovaries; in contrast, the number of MOF per ovary was increased more than 4 times (Fig. 5A–D).

To further investigate the late impacts of embryonic ATZ exposure on molecular mechanisms implicated in follicle growth and maturation, we performed a quantitative analysis of genes involved in oocyte development by qPCR in 6-d.o. ovaries. We found that embryonic exposure to ATZ leads to a decrease in the expression of genes associated with the oocyte growth signaling pathway, including *Sohlh2* and *Foxo3* genes^38, 39^. The RNA level of *Smad4*, which encodes a protein associated with granulosa cell signaling function, was also decreased. Similarly, the RNA level for the *Kit* gene, which encodes the protein involved in meiotic arrest as well as the growth and differentiation of theca cells^40^, was downregulated (Fig. 5E). ATZ-exposed ovaries also had lower levels of *Inha* and *Inhba* transcripts, both of which are normally expressed in granulosa cells in early developing follicles during the first maturation wave^41^.

In addition, to assess whether ATZ can affect detoxifying enzymes, we analyzed the expression of the most common oxidative stress responsive genes *Sod1* and *Gpx1* in 6-d.o. ovaries. We found that embryonic exposure to ATZ causes a significant decrease in the expression of both genes, suggesting their role in inducing oxidative stress (Suppl. Fig. 2). Thus, our data show that embryonic exposure to ATZ decreases the expression of the genes associated with follicle growth and maturation and genes encoding detoxifying enzymes in 6-d.o. ovaries.

### Embryonic exposure to ATZ does not significantly affect the level of steroid hormones in the adult ovary

To investigate whether the embryonic effects of ATZ affect maturation and puberty in adult mice, we analyzed the day of the opening of the vaginal cavity in young animals. This parameter reflects the beginning of the first mouse estrus. We found that *in utero* exposure to ATZ leads to delayed vaginal opening in female mice for 1.24 days (n = 15; p = 0.0403, t-test) without altering the body and ovary weights (Suppl. Fig. 3A–C). Because the vaginal opening can be influenced by changes in steroid hormone levels, we performed the analysis of hormones in the serum. Our analysis showed that the levels of circulating estradiol (E2) displayed a slight but not significant decrease in ATZ-exposed mice compared to control animals (n = 15). The levels of P4, pregnenolone (Preg), and LH were also not significantly altered (Suppl. Fig. 3D,E). These data suggest that embryonic exposure to ATZ delays puberty; however, the contribution of steroid hormones is unlikely to be significant.

### The number of primordial follicles is decreased and the number of MOFs is increased in ATZ-exposed animals

**Figure 6:**
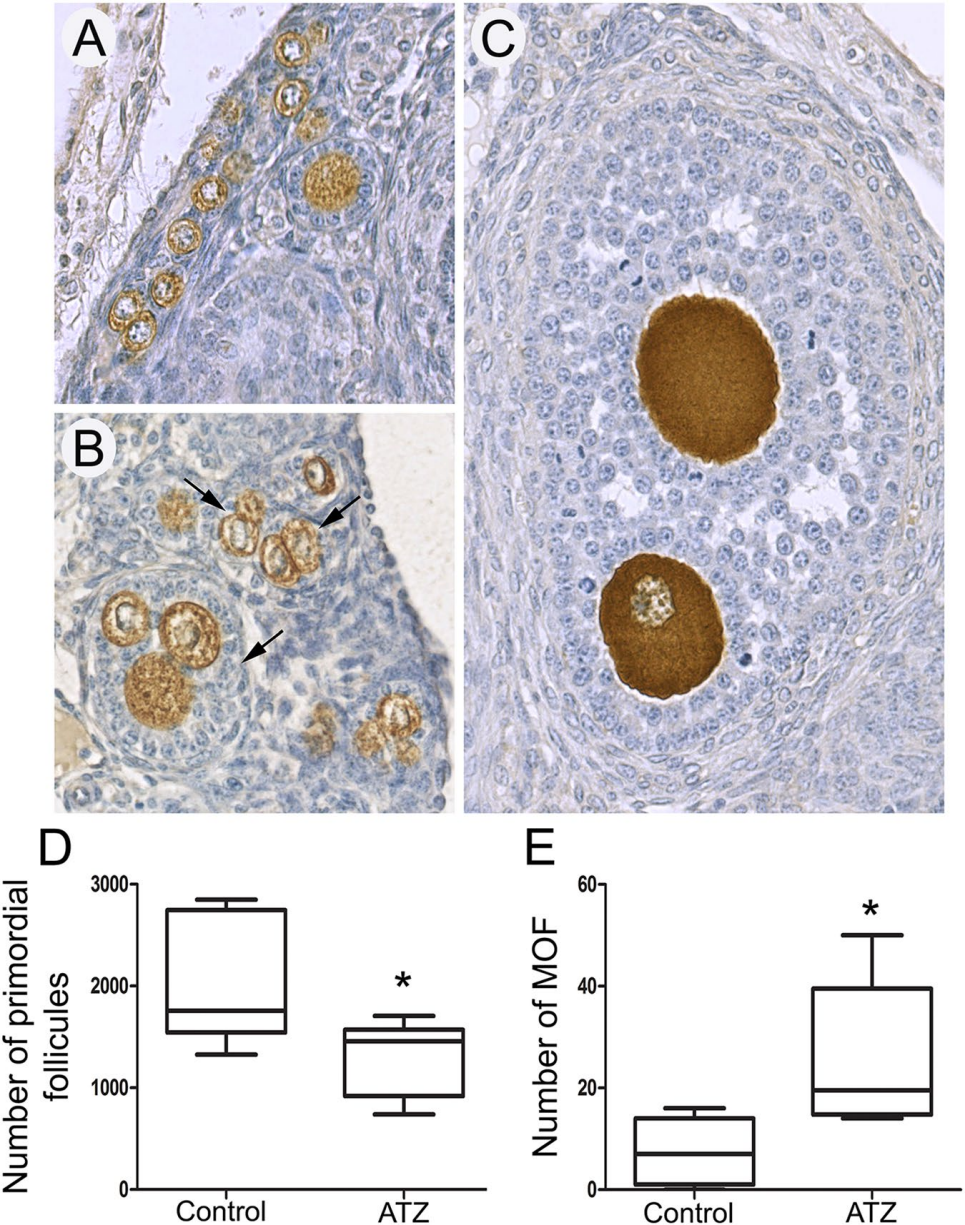
*In utero* exposure to Atrazine disturbs folliculogenesis in young adults on the day of vaginal opening. Histological sections of the adult ovaries of (**A**) control and (**B**,**C**) ATZ-treated mice. (**B**) Primary, secondary and (**C**) antral follicles containing two to four oocytes were often observed in ATZ-treated samples (arrows). Quantification of the number of primordial (**D**) and multi-oocyte (**E**) follicles in control and ATZ-treated ovaries. (n = 7; *p ≤ 0.05, nonparametric Mann-Whitney test). The data are presented as median values, and the lower and upper quartile values are shown.

To analyze the effects of ATZ exposure on follicular development in adult females, we performed a quantitative analysis of all follicle types in serial sections of 4-5-week-old ovaries at first estrus (Fig. 6). We found a significant reduction of 23% in the number of primordial follicles in ATZ-exposed mice (p = 0.035, nonparametric Mann-Whitney test). We also detected an increase of nearly 4-fold in MOF numbers in the ATZ-treated animals compared to controls. Our observed effect in 4–5-week-old ovaries was similar to the increase in MOFs observed in 6-d.o. ovaries (Figs 5C and 6E and data not shown). To reveal how embryonic exposure to ATZ affects the reproductive aging of exposed females, we also examined sections from one-year-old mice. We found that in one year-old ATZ–exposed female mice, the weights of the ovaries were significantly lower, and the number of advanced follicles was also lower compared to controls. We observed *corpora lutea* in control and ATZ-treated ovaries, suggesting that in control and ATZ-exposed ovaries, the endocrine activity is comparable (Suppl. Fig. 4). Our data demonstrate that embryonic exposure to ATZ affects follicular development and reduces the number of follicles in aging mice.

## Discussion

We previously showed that exposure to ATZ disrupts meiosis in male mice^18^. The aim of the present study was to investigate whether prenatal exposure to ATZ also affects female meiosis and whether ATZ exposure during the early developmental program will impact oocyte maturation in the adult stage. We analyzed major meiotic events, which occur during embryonic development, and we investigated the effects of ATZ on ovarian folliculogenesis after birth, at puberty and in aging female mice.

### Embryonic exposure to ATZ impacts the key events of meiotic prophase

Here, we show that in ATZ-exposed mice, there is an increase in DMC1 foci in the *zygotene* stage of meiotic oocytes. The formation of DSBs is required for the initiation of the homologous chromosome search to segregate them before the first meiotic division. The cell-to-cell variation in the number of DMC1 foci is high in spermatocytes, with the early foci numbers being more variable than later foci numbers^42^. The large variation in DMC1 foci reflects the dynamics of DSB formation and repair. The most likely explanation for an increase in DMC1 foci is inefficient DNA repair under toxic conditions. Specifically, previous studies have shown that exposure to ATZ induces oxidative stress in exposed cells, which is characterized by the increased activity of the cytochrome C system and detoxifying glutathione S-transferases^43, 44^. Oxidative stress increases the concentration of oxidizing molecules known as reactive oxygen species (ROS). The increase in ROS could increase DNA damage^45^ and interfere with DNA DSB repair mechanisms^46^. It was demonstrated that under toxic conditions, the repair of DSBs becomes inefficient and slows down (see ref. 47 for the review). In the worm *Caenorhabditis elegans*, BPA exposure results in impaired chromosome synapsis and the disruption of meiotic DSB repair progression^48^. If DSBs persist, the check point mechanisms inhibit meiosis to prevent its progression, and meiotic arrest can lead to cells being eliminated^49^.

Our results similarly demonstrate that exposure to ATZ not only delays meiotic synapsis but also affects the number of crossovers. A reduced number of crossovers has been found in infertile men^50, 51^. It was suggested that crossover formation is strictly regulated to avoid aneuploidy^42^. There is substantial genetic variability in crossover numbers between different mouse strains^52^ and between sexes^53^, suggesting the existence of mechanisms that control crossover homeostasis. The decrease in crossover number in ATZ-exposed oocytes suggests that the mechanisms controlling the crossover number become affected upon ATZ exposure. However, whether meiotic changes can directly promote aneuploidy or meiotic arrest remains to be determined.

### Oxidative stress in females affects meiosis

It has been shown that exposure to ATZ causes oxidative stress in male rats^54^ and mice^18^ and is associated with the increased production of reactive oxygen species (ROS) in the serum^18^. Embryonic exposure to ATZ in mice promotes transgenerational effects, and in third-generation males, the genes encoding proteins for mitochondrial function are downregulated^31^, suggesting that mitochondria are one of the main targets of ATZ.

Here, we show decreased expression levels of genes encoding the detoxifying enzymes *Sod1* and *Gpx1*. Our results are consistent with previous experiments in rats, where ATZ exposure causes a significant decrease in the activities of both enzymes^54^. The alteration in the detoxifying enzyme activities suggests that ATZ-treated animals are continuously exposed to ROS. We suggest that oxidative stress could cause inefficient DNA repair during meiosis. Specifically, we show that 8-oxo-G staining is increased in the nuclei of ATZ-treated oocytes. It is suggested that the production of 8-oxo-G is associated with mutations and could lead to diseases^55^. Unlike nuclear DNA, mitochondrial DNA is highly exposed to local ROS due to its proximity to the oxidative phosphorylation machinery^56^. Importantly, it was shown that the repair of 8-oxo-G is also more efficient in mitochondrial DNA than in nuclear DNA^57^, suggesting that nuclear DNA damage in ATZ-exposed subjects could lead to delays in DNA repair due to the necessity of repairing 8-oxo-G nucleotides. We suggest that meiotic events could also be affected by the availability of ATP. Previous reports have demonstrated that ATP synthase is a major target of ATZ^58^, suggesting that the exposure to ATZ is also accompanied with a decrease in ATP, the cellular source of energy. ATP energy is required for a large number of physiological functions, and it is mostly produced in mitochondria through oxidative phosphorylation. Importantly, most DNA repair proteins involved in meiosis are ATP-dependent. We analyzed the genes annotated in the Amigo Gene ontology database as associated with “meiosis” and “ATP binding”. We identified 149 “meiosis” and 1,372 “ATP-binding” genes. Importantly, 25% of genes encoding “meiosis’ proteins are annotated as “ATP-binding”. These genes are involved in the early step of homologous recombination, and they include *Spo11, Atm, Rad51, Dmc1* and genes encoding proteins that are essential for crossover formation, such as the *Msh5 and Mlh1* genes. The reduced production of ATP suggests that ATZ exposure lowers the efficiency of the processes that require ATP.

### ATZ exposure disrupts the follicle stockpile

The formation of ovarian follicles, which occurs after birth in rodents, is a complex process involving interactions between growth factors and signaling pathways and is controlled by a meiotic clock^59, 60^. Although we did not observe any decrease in germ cell numbers at embryonic stage E18.5, the number of primordial follicles was decreased in young adult females. Recent work demonstrates that exposure to chemicals such as phthalate^61^ or to cigarette smoke in adults^62^ leads to a decreased follicle stockpile. We also found that embryonic exposure to ATZ leads to an increase in the rate of MOF incidence in 6-d.o. and adult females. Our data are consistent with the previously published study, where rare MOFs were observed in Wistar female rats that were exposed to ATZ during adulthood^19^. It was previously proposed that MOFs are formed as a result of the breakdown of incomplete oocyte cysts, which is a normal process^63^, or by the fusion of two distinct oocytes after birth^64^. Cyst breakdown is developmentally programmed and occurs synchronously in mice approximately at E20.5, just before the onset of primordial follicle formation^60^. We show that embryonic exposure to ATZ alters the signaling pathways involved in cyst breakdown and leads to the appearance of MOFs after birth. Since cyst breaking is necessary to give rise to primordial follicles, the appearance of MOFs could explain the reduced number of primordial follicles in ATZ-treated ovaries.

We found several changes in the expression of ovarian factors in 6-d.o. mice. On one hand, we observed decreased expression of several oocyte markers, including *Kit*, *Foxo3* and *Sohlh2*, but not *Sohlh1*, *Fila*, *Nobox*, *Gdf9* and *Lhx8* in 6-d.o. ATZ-exposed mice.

Specifically, *Foxo3* mRNA levels were reduced after treatment. *Foxo3* knockout leads to premature ovarian failure (POF) in mice^39^. POF is a common cause of infertility, but the majority of cases remain idiopathic. We believe that embryonic exposure to ATZ alters the expression of genes that are critical for follicular development (e.g., *Foxo3*) and thus leads to a subsequent decrease of the developing pool of primordial follicles in adults. We believe that although meiotic progression might not be essential for follicle assembly^65^, the adverse changes in signaling and transcriptional factors might influence the viability of the resulting primordial follicles by affecting multiple processes.

Ovaries deficient in either *Sohlh2* or *Foxo3a* display abnormal primordial to primary follicle transition with either an absence of proliferation in granulosa cells or a global activation of proliferation, leaving the ovary empty by the age of 15 weeks^38, 39^. In addition, *Sohlh2* deficiency is associated with decreased expression of *Sohlh1, Lhx8, Nobox, Fogla, Zp1, Zp3, Gdf9*, and *Pou5f1* and increased expression of *Stra8* at day of birth^66^. KIT signals through multiple pathways, including PI3K^67^. Inactivation of *Kit* signaling via the PI3K pathway results in decreased primordial follicle survival, an abnormal accumulation of ovarian follicles arrested at an early stage of maturation, and abnormal accumulation of FOXO3 protein within the oocyte nucleus^68^. The transcriptional decrease in *Kit*, *Foxo3* and *Sohlh2* expression that we observed in 6-d.o. ovaries may be associated with an alteration of primordial to primary follicle activation and the primary to secondary follicle progression that may lead to an early depletion of the primordial follicle stockpile in ATZ-exposed mice.

On the other hand, we found decreased levels of the granulosa cell markers *Inha*, *Inhba*, and *Smad* 4 with unaltered expression of *Foxl2*. Strikingly, these 3 impacted genes encode proteins of the TGF beta signaling pathway, which is crucial for granulosa cell growth and differentiation and is important for steroidogenesis. Although *Inha (inhibin A)* and *Inhba* (also known as activin B) were initially identified as genes that control FSH production, their role in early folliculogenesis has now been established. *Inha* knockout mice exhibit numerous multilayered follicles that are far more advanced than those observed in age-matched controls^69^. The expression pattern of *Inha* is consistent with a decreased primordial pool of oocytes at first estrus, suggesting that ovaries may progress faster, thereby depleting the primordial follicles. Inhba/activin B is an important interactor of estrogens in the early mouse ovary^70^. Activin is also capable of inducing the expression of ERs in the mouse ovary, suggesting the existence of important interplays between activin and estrogen signaling^71^. *Smad4* conditional knock-out animals exhibit abnormal, small follicles with luteinizing cells surrounding either oocytes or the oocyte remnants^72^. Altogether, the alterations in gene expression of either the folliculogenesis-related oocyte or granulosa cell markers that we observed in 6-d.o. ATZ-treated mice may lead to the abnormal folliculogenesis that we observed later in young adult females. We believe that embryonic exposure to ATZ alters the expression of genes that are critical for follicular development and thus leads to a subsequent decrease of the developing pool of primordial follicles in the adult. Although the meiotic progression might not be essential for follicle assembly^65^, the changes in signaling and transcriptional factors might influence the viability of the resulting primordial follicles by affecting multiple processes.

### In utero exposure to ATZ leads to long-term effects on female reproductive physiology

We found a delay in vaginal opening in ATZ-treated animals. Our finding is consistent with a recent study that also reported the delay of vaginal opening in rats exposed to ATZ either during embryonic or post-natal development^16, 17, 73, 74^. Altogether, our data suggest that exposure to ATZ during the developmental period leads to a delay in reproductive maturity or the onset of puberty in mice, a reduction in primordial oocyte numbers and a decrease in oocyte stockpiles in adulthood. We cannot, however, exclude the possibility that the defects in puberty onset could be additionally triggered by changes in the central nervous system caused by ATZ exposure^16, 17, 73^. In summary, our results demonstrate that embryonic exposure to ATZ leads to profound defects in meiosis, affects oocyte maturation, alters normal follicular development and reduces the reproductive lifespan. Our data provide an important insight into the negative effects of ATZ on the female reproductive system.

## Materials and Methods

### Ethics statement

The animal facility used for the present study is licensed by the French Ministry of Agriculture (agreement D35–238–19). All animal procedures were performed according to the Ethics Committee of the Ministry of the Research of France (number of agreement: 01861.02). All experimental procedures followed the ethical principles outlined in the Ministry of Research Guide for Care and Use of the Laboratory Animals and were approved by the local Animal Experimentation Ethics Committee (C2EA-07).

### Animal treatment

The outbred Swiss (RjOrl) mice were used for all experiments. The day of vaginal plug detection was considered as embryonic day 0.5 (E0.5). ATZ (Sigma Aldrich, 45330) was administered via oral gavage to 6–8-week-old pregnant female mice from E6.5 to E15.5. The herbicide was suspended in olive oil and administered as 100 mg/kg/day in a volume of 150 μl. The control mice were treated with the same volume of oil. Control and ATZ-treated pregnant females were sacrificed via euthanasia with CO2, and embryos were removed and sacrificed by decapitation. Embryonic gonads were either fixed or kept at −80 °C until use. For each experiment, 4 to 6 embryos from at least three different litters were used. To analyze the ovaries of adult female mice, they were anesthetized with ketamine/xylazine solution and sacrificed by cardiac exsanguination. Young adult females were sacrificed on the day of vaginal opening, and at the first estrus, one-year-old females were put with males and sacrificed on the day of vaginal plug detection.

### Hormone measurement

The serum was collected from ketamine/xylazine-anesthetized females at the day of the opening of the vaginal cavity (vaginal opening) by terminal cardiac exsanguination, and aliquots were stored at −80 °C. Progesterone (P), pregnenolone (Preg) and estradiol (E2) were assayed in two steps using mass spectrometry coupled with gas chromatography^37^. Briefly, sera were overloaded with deuterated steroid internal standards (CDN isotopes Inc., Canada) and extracted with 1-chlorobutane. The organic extracts were purified on conditioned LC-Si SPE columns (Varian, France). E2 was derivatized with pentafluorobenzoyl chloride (103772–1 G, Sigma Aldrich). P and Preg were derivatized with heptafluorobutyric anhydride (HFBA) (H1006, Sigma Aldrich). The final extracts were reconstituted in isooctane and transferred into conical vials for injection into the GC system (6890 N, Agilent Technologies, Massy, France) using 50% phenyl methylpolysiloxane VF-17MS capillary columns (20 m × 0.15 mm, internal diameter, 0.15 µm film thickness) (Varian, Les Ulis, France). An HP5973 (Agilent Technologies, Massy, France) quadrupole mass spectrometer equipped with a chemical ionization or an electron impact source and operating in the single-ion monitoring (SIM) mode was used for detection.

LH measurements were performed with a specific ELISA kit according to the manufacturer’s instructions (KA2332, Abnova, Walnut CA, USA). In each hormone measurement, the data from at least 15 treated and control animals were averaged and plotted, and the results were expressed as a mean hormone level value in ng/ml +/− SD.

### Preparation and immunostaining of structurally preserved nuclei

Structurally preserved nuclei (SPN) for three-dimensional analysis were prepared by mincing fresh or frozen E13.5 and E15.5 ovarian tissue in DMEM medium (Life Technologies, GIBKO) and 0.5% protease inhibitor at 4 °C. The ovary suspensions were mixed with equal amounts of 3.7% (vol/vol) paraformaldehyde and 0.1 M sucrose, pH 7.2, and placed on glass slides. After air drying at room temperature, slides were washed 4 times for 1 min each in 0.4% (v/v) Kodak Photo-Flo solution, air-dried and kept at −80 °C until further use. For the quantitative analysis of 8-oxo-G, we performed immunostaining against this DNA adduct according to protocol similar to immunostaining of ovarian spreads. SPN were immunostained against mouse monoclonal 8-oxo-G (MAB, 3560, MerckMillipore) and rabbit polyclonal RPA (SC-25376) antibodies. We analyzed 4 independent biological replicates and at least 90 cells total for each condition. We separated the staining according to the pattern (preferential nuclear or preferential cytoplasmic staining), and the data were plotted and represented as a proportion of the cytoplasmic or nuclear fractions.

### Meiotic chromosome spreads

The ovaries were dissected at E15.5, E16.5 and E18.5 and kept in ice-cold PBS. The ovaries were placed in a 20 µl drop of 100 mM sucrose, disrupted with tweezers and pipetted up and down with small tips until a cell suspension cell was formed. The cells were transferred onto a glass slide with 100 µl of 1% paraformaldehyde containing 0.1% (v/v) Triton X-100 and were kept for 2–4 hours in a humidified chamber at RT, and then, they were dried. The slides were washed 4 times for 1 min each in 0.4% (v/v) Kodak Photo-Flo solution; then, they were air-dried and kept at −80 °C until use.

### Immunolabeling on ovarian spreads

The ovarian spreads were blocked with blocking buffer (0.1% (v/v) donkey serum, 0.03% (w/v) BSA, and 0.005% (v/v) Triton X-100 in PBS) for 20 min at 37 °C in a humidified chamber. The slides were incubated with a primary antibody diluted in blocking solution for 1 hour at 37 °C in a humidified chamber, washed three times with 0.4% (v/v) Kodak Photo-Flo/PBS and incubated with a secondary fluorescent Alexa antibody diluted 1:500 for 20 min at RT. The slides were washed three times with 0.4% (v/v) Kodak Photo-Flo/PBS and mounted with Vectashield mounting media containing 5 µg/ml DAPI (Vector Laboratories, Burlingame, CA). The slides were analyzed with an epifluorescence Axio Imager M1 microscope (ZEISS, France), and the pictures were taken using a 63X objective with AxioVision Rel 4.7.1 (Zeiss, France). For quantitative assays, pictures of at least 100 cells were taken and quantitatively analyzed using Photoshop. For DMC1 foci visualization, the slides from E15.5 were incubated with primary goat anti-DMC1 (1:50) (Santa Cruz, sc-8973) and mouse anti-SYPC3 (1:100) (Santa Cruz, sc-33195) antibodies. DMC1 foci were counted in at least 100 cells at the zygotene stage in control or ATZ-treated samples.

For the quantification of crossover numbers, E18.5 ovarian spreads from control or ATZ-treated samples were immunostained with primary mouse anti-MLH1 (1:20) (Cell Signaling, 4C9C7) and rabbit anti-SYPC3 (1:100) (Santa Cruz, sc-33195) antibodies. MLH1 foci were counted in at least 100 cells at the pachytene stage in control or ATZ-treated samples.

For the quantitative analysis of synapsis efficiency, E16.5 ovary spreads were immunostained with antibodies against mouse anti-SYPC3 (1:100) and rabbit anti-SYCP1 (1:50) (Abcam, ab15087). A total of at least 100 meiotic cells were selected at random and grouped according to the following criteria: the homologous chromosomes were either fully synapsed (100% overlap between SYCP1 and SYCP3) or at least one bivalent exhibited incomplete synapsis.

### Immunohistochemistry

For the analysis of tissue morphology, E18.5 embryonic ovaries from the control and ATZ-treated groups were fixed in Bouin’s solution for 16 hours, dehydrated and embedded in paraffin. Whole organs were cut, and every 5^th^ section (5-μm thick) was mounted on a slide. In total, ovaries from 5 animals of each group were sectioned for the analysis. The sections were deparaffined, rehydrated and stained with hematoxylin and eosin. To analyze the number of embryonic ovarian germ cells, the ovaries from control and ATZ-treated groups were fixed in 4% (w/v) PFA solution for 16 hours, dehydrated and embedded in paraffin. The sections were deparaffined and rehydrated, and the epitopes were unmasked in 0.01 M citrate buffer, pH 6 at 80 °C for 45 min. After washing in 1X PBS-0.05% Tween (PBS-T), the sections were incubated with an anti-DDX4 (VASA) (ab13840, Abcam) antibody (1:500) in PBS-T overnight at 4 °C in a humidified chamber. After washing in PBS-T, the sections were incubated with a fluorescent secondary anti-rabbit antibody (1:500) for 1 hour in a humidified chamber at room temperature. The sections were counterstained with 0.001% (v/v) 4,6-diamidino-2-phenylindole dihydrochloride (DAPI) and mounted in Vectashield solution. Tissue preparations from adult ovaries were performed as described above for the embryonic samples. After washing in 1X PBS, sections were placed in 1% H_2_O_2_-PBS for 5 min at 37 °C. The sections were blocked with 2% (w/v) BSA diluted in 1X PBS-T (Tween 20, 0.05%) for 20 min at RT. The sections were washed and incubated with anti-MSY2 antibody (Santa Cruz, sc-21316) (1:400 in PBS/2% (w/v) BSA) overnight at 4 °C in a humidified chamber. After washing in PBS-T, the sections were incubated with biotinylated anti-goat antibody (1:500) for 1 hour in a humidified chamber, washed with PBS-T and incubated with the ABC kit (Vector Laboratories, CA, USA) for 30 min at RT. Immunostaining was developed with DAB solution (D3939, Sigma, MI, USA) for 5 min. The tissue sections were counterstained with Masson’s hemalun solution. Images of the whole slide were captured using the NanoZoomer Digital Pathology (NDP) System C9600 with the NDPScan 2.5 software.

### Quantification of germ cells and follicles

Germ cells in E18.5 ovaries were quantified using images taken with an AxioImager microscope equipped with an AxioCam MRc5 camera and the AxioVision software version 4.8.2 (Zeiss, Le Pecq, France) using a 20X objective lens. To quantify the number of DDX4-positive cells, at least 6 different sections in 6 different replicates were scored. The total number of DDX4-positive cells was counted on each section, and it was reported as a ratio to the surface area of the section, which estimated using the ImageJ software. The follicles were quantified using the NDPview software. Quantification was facilitated by YBS2 labelling, and follicles were counted when the nuclei of the oocytes were visible in the follicle. The follicles were classified as primordial when the oocyte was surrounded by a single layer of flattened granulosa cells; as primary, when the granulosa cells of the single layer became cuboidal; as secondary/preantral, when the number of granulosa cell layers exceeded one; and as antral, when the antrum cavity appeared. The follicles were classified as atretic when they contained at least 2 pycnotic granulosa cells. MOFs were defined as two or more oocytes that were surrounded by a layer of granulosa cells. At least five ovaries from control and ATZ-exposed mice were used for quantitative analysis.

### RNA extraction and quantitative PCR

Total RNA was extracted using the RNeasy plus mini kit (Qiagen) according to the manufacturer’s instructions. Reverse transcription was performed with 1 µg of RNA using the iScript™ cDNA Synthesis Kit according to the manufacturer’s instructions (Bio-Rad). The resulting cDNA was diluted 5 times and used for quantitative PCR. The primer sequences used for qPCR are indicated in Table 1. qPCR was performed using the iTaq Universal SYBR Green Supermix (Bio-Rad) according to the manufacturer**’**s instructions on a CFX384 Touch Real-Time PCR Detection system (Bio-Rad). The gene copy number was calculated with Bio-Rad CFX Manager 3.1. PCR amplification of the coding regions of *ActB* and *Rplp0* was used for normalization. The data from at least 6 samples were analyzed, compared, plotted and expressed as a fold change in treated samples compared to controls.

**Table 1:** Primers used in quantitative RT-PCR.

Genes	Accession no.	Primer sequence 5′ to 3′	Position (nt)	Size (nt)
*ActB*	NM_007393.5	5′-CCAACTGGGACGACATGGAG-3′	339–530	192
		5′-ACAGCACAGCCTGGATGGC-3′		
*Rplp0*	NM_007475	5′-ACCCTGAAGTGCTCGACATC-3′	720–908	208
		5′-AGGAAGGCCTTGACCTTTTC-3′		
*Figla*	NM_012013.2	5′-GGTGCCACAGAATACATACAGA-3′	361–523	163
		5′-CACAGCTGGTAGGTTGGGTA-3′		
*Foxl2*	NM_012020.2	5′-GCAAGGGAGGCGGGACAACAC-3′	158–312	155
		5′-GAACGGGAACTTGGCTATGATGT-3′		
*Foxo3*	XM_006512806.1	5′-CAAACGGCTCACTTTGTCCC-3′	855–1007	153
		5′-TCATTCTGAACGCGCATGAA-3′		
*Gdf9*	XM_006532220.2	5′-CAAACCCAGCAGAAGTCACC-3′	395–593	199
		5′-GGAGGAAGAGGCAGAGTTGT-3′		
*Lhx8*	NM_010713	5′-GGGGAAGAAGGACTGGTGAA-3′	390–534	249
		5′-TTGTCCACAATCTCCAGGCC-3′		
*Inha*	NM_010564.4	5′-GGCGTCTGCCTCGAAGACAT-3′	344–532	189
		5′-GTTGGGATGGCCGGAATACA-3′		
*Inhba*	XM_006516558.2	5′-CAGGAGGGCCGAAATGAATG-3′	2216–2413	198
		5′-CGGATGGTGACTTTGGTCCTG-3′		
*Kit*	NM_001122733.1	5′-AGCGTCTTCCGGCACAACGG-3′	1515–1658	144
		5′-GCCAATGAGCAGCGGCGTGA-3′		
*Nobox*	XM_006505717.2	5′-GCTGGAAGAACTGGAGAGGA-3′	717–900	184
		5′-GGCTGCAGGACCATTCTTAG-3′		
*Smad4*	XM_011246855.1	5′-TAATCGCGCATCAACGGAGA-3′	769–958	190
		5′-CTGCTGCTGTCCTGGCTGAG-3′		
*Sohlh1*	NM_001001714.1	5′-GGGCCAATGAGGATTACAGA-3′	76–194	119
		5′-CACAGGAGCTGTGCAGAGAG-3′		
*Sohlh2*	NM_028937.3	5′-TCAGTGAGCCGCTGACCTTG-3′	382–531	150
		5′-AAAAACGCCCTCCGAGTTCAC-3′		
*Sod1*	NM_011434.1	5′-GGACAATACACAAGGCTGTACCA-3′	272–384	113
		5′-CAGTCACATTGCCCAGGTCTC-3′		
*Gpx1*	NM_008160.6	5′-TCTCTGAGGCACCACGATCC-3′	368–492	125
		5′-TCTTGCCATTCTCCTGGTGTC-3′		

### Statistical analysis

Statistical analyses were conducted using the GraphPad Prism 6 software. We compared the number of embryonic DDX4-positive cells, the number of DMC1, the number of MLH1 foci, the levels of LH and steroid hormones using two-tailed Student’s unpaired t-tests. We compared the number of synapsed and unsynapsed chromosomes, the number of the different types of follicles, qPCR data of ovarian gene expression, the ovary and body weights and the time of vaginal opening using the Mann-Whitney statistical test. Results were considered to be statistically significant when the p-value was ≤0.05.

## Electronic supplementary material

Supplemental figures

## References

[1] Weidner IS, Moller H, Jensen TK, Skakkebaek NE (1998). Cryptorchidism and hypospadias in sons of gardeners and farmers. Environ Health Perspect.

[2] Martin RH (2006). Meiotic chromosome abnormalities in human spermatogenesis. Reprod Toxicol.

[3] Multigner L (2010). Chlordecone exposure and risk of prostate cancer. Journal of clinical oncology: official journal of the American Society of Clinical Oncology.

[4] Cohn BA, Wolff MS, Cirillo PM, Sholtz RI (2007). DDT and breast cancer in young women: new data on the significance of age at exposure. Environmental health perspectives.

[5] Kahl, V. F. *et al*. Telomere measurement in individuals occupationally exposed to pesticide mixtures in tobacco fields. *Environmental and molecular mutagenesis* (2015). 10.1002/em.21984 26426910

[6] Toccalino, P. L., Gilliom, R. J., Lindsey, B. D. & Rupert, M. G. Pesticides in Groundwater of the United States: Decadal-Scale Changes, 1993–2011. *Ground water* (2014). 10.1111/gwat.12176 24597577

[7] Kock-Schulmeyer M (2014). Four-year advanced monitoring program of polar pesticides in groundwater of Catalonia (NE-Spain). The Science of the total environment.

[8] Woudneh MB, Ou Z, Sekela M, Tuominen T, Gledhill M (2009). Pesticide multiresidues in waters of the Lower Fraser Valley, British Columbia, Canada. Part II. Groundwater. Journal of environmental quality.

[9] Vonberg D (2014). Atrazine soil core residue analysis from an agricultural field 21 years after its ban. Journal of environmental quality.

[10] Nodler K, Licha T, Voutsa D (2013). Twenty years later–atrazine concentrations in selected coastal waters of the Mediterranean and the Baltic Sea. Marine pollution bulletin.

[11] Vonberg D (2014). 20 years of long-term atrazine monitoring in a shallow aquifer in western Germany. Water research.

[12] Chevrier C (2011). Urinary biomarkers of prenatal atrazine exposure and adverse birth outcomes in the PELAGIE birth cohort. Environ Health Perspect.

[13] Ochoa-Acuna H, Frankenberger J, Hahn L, Carbajo C (2009). Drinking-water herbicide exposure in Indiana and prevalence of small-for-gestational-age and preterm delivery. Environmental health perspectives.

[14] Victor-Costa AB, Bandeira SM, Oliveira AG, Mahecha GA, Oliveira CA (2010). Changes in testicular morphology and steroidogenesis in adult rats exposed to Atrazine. Reproductive toxicology.

[15] Song Y, Jia ZC, Chen JY, Hu JX, Zhang LS (2014). Toxic effects of atrazine on reproductive system of male rats. Biomedical and environmental sciences: BES.

[16] Ashby J, Tinwell H, Stevens J, Pastoor T, Breckenridge CB (2002). The effects of atrazine on the sexual maturation of female rats. Regulatory toxicology and pharmacology: RTP.

[17] Davis LK (2011). The effects of prenatal exposure to atrazine on pubertal and postnatal reproductive indices in the female rat. Reproductive toxicology.

[18] Gely-Pernot A (2015). The epigenetic processes of meiosis in male mice are broadly affected by the widely used herbicide atrazine. BMC genomics.

[19] Juliani CC, Silva-Zacarin EC, Santos DC, Boer PA (2008). Effects of atrazine on female Wistar rats: morphological alterations in ovarian follicles and immunocytochemical labeling of 90 kDa heat shock protein. Micron.

[20] Cooper RL, Stoker TE, Tyrey L, Goldman JM, McElroy WK (2000). Atrazine disrupts the hypothalamic control of pituitary-ovarian function. Toxicol Sci.

[21] McMullin TS (2004). Evidence that atrazine and diaminochlorotriazine inhibit the estrogen/progesterone induced surge of luteinizing hormone in female Sprague-Dawley rats without changing estrogen receptor action. Toxicological sciences: an official journal of the Society of Toxicology.

[22] Foradori CD (2014). The effect of atrazine administered by gavage or in diet on the LH surge and reproductive performance in intact female Sprague-Dawley and Long Evans rats. Birth defects research. Part B, Developmental and reproductive toxicology.

[23] Eldridge JC (1994). Short-term effects of chlorotriazines on estrus in female Sprague-Dawley and Fischer 344 rats. Journal of toxicology and environmental health.

[24] Qin L (2015). Atrazine triggers developmental abnormality of ovary and oviduct in quails (Coturnix Coturnix coturnix) via disruption of hypothalamo-pituitary-ovarian axis. Environmental pollution.

[25] Gojmerac T (1996). Serum biochemical changes associated with cystic ovarian degeneration in pigs after atrazine treatment. Toxicology letters.

[26] Hochberg Z (2011). Child health, developmental plasticity, and epigenetic programming. Endocr Rev.

[27] Susiarjo M, Hassold TJ, Freeman E, Hunt PA (2007). Bisphenol A exposure in utero disrupts early oogenesis in the mouse. PLoS genetics.

[28] Di Giacomo M (2005). Distinct DNA-damage-dependent and -independent responses drive the loss of oocytes in recombination-defective mouse mutants. Proceedings of the National Academy of Sciences of the United States of America.

[29] Arnheim N, Calabrese P, Tiemann-Boege I (2007). Mammalian meiotic recombination hot spots. Annu Rev Genet.

[30] Roig I (2004). Female-specific features of recombinational double-stranded DNA repair in relation to synapsis and telomere dynamics in human oocytes. Chromosoma.

[31] Hao, C. *et al*. Exposure to the widely used herbicide atrazine results in deregulation of global tissue-specific RNA transcription in the third generation and is associated with a global decrease of histone trimethylation in mice. *Nucleic acids research* (2016). 10.1093/nar/gkw840 PMC5175363 27655631

[32] Jin, Y. *et al*. Oral exposure of pubertal male mice to endocrine-disrupting chemicals alters fat metabolism in adult livers. *Environmental toxicology* (2014). 10.1002/tox.22013 24916741

[33] Trentacoste SV, Friedmann AS, Youker RT, Breckenridge CB, Zirkin BR (2001). Atrazine effects on testosterone levels and androgen-dependent reproductive organs in peripubertal male rats. Journal of andrology.

[34] Scialli AR, DeSesso JM, Breckenridge CB (2014). Developmental toxicity studies with atrazine and its major metabolites in rats and rabbits. Birth defects research. Part B, Developmental and reproductive toxicology.

[35] Maga G (2008). Replication protein A and proliferating cell nuclear antigen coordinate DNA polymerase selection in 8-oxo-guanine repair. Proceedings of the National Academy of Sciences of the United States of America.

[36] Maga G (2007). 8-oxo-guanine bypass by human DNA polymerases in the presence of auxiliary proteins. Nature.

[37] Chalmey C (2013). Systemic compensatory response to neonatal estradiol exposure does not prevent depletion of the oocyte pool in the rat. PloS one.

[38] Choi Y, Yuan D, Rajkovic A (2008). Germ cell-specific transcriptional regulator sohlh2 is essential for early mouse folliculogenesis and oocyte-specific gene expression. Biology of reproduction.

[39] Castrillon DH, Miao L, Kollipara R, Horner JW, DePinho RA (2003). Suppression of ovarian follicle activation in mice by the transcription factor Foxo3a. Science.

[40] Thomas FH, Vanderhyden BC (2006). Oocyte-granulosa cell interactions during mouse follicular development: regulation of kit ligand expression and its role in oocyte growth. Reproductive biology and endocrinology: RB&E.

[41] Lovell TM, Gladwell RT, Groome NP, Knight PG (2003). Ovarian follicle development in the laying hen is accompanied by divergent changes in inhibin A, inhibin B, activin A and follistatin production in granulosa and theca layers. The Journal of endocrinology.

[42] Cole F (2012). Homeostatic control of recombination is implemented progressively in mouse meiosis. Nature cell biology.

[43] Abarikwu SO, Farombi EO, Pant AB (2012). Kolaviron biflavanoids of Garcinia kola seeds protect atrazine-induced cytotoxicity in primary cultures of rat Leydig cells. International journal of toxicology.

[44] Jin Y (2014). Exposure of mice to atrazine and its metabolite diaminochlorotriazine elicits oxidative stress and endocrine disruption. Environmental toxicology and pharmacology.

[45] da Silva J (2016). DNA damage induced by occupational and environmental exposure to miscellaneous chemicals. Mutation research.

[46] Mehta A, Haber JE (2014). Sources of DNA double-strand breaks and models of recombinational DNA repair. Cold Spring Harb Perspect Biol.

[47] Jackson SP (2002). Sensing and repairing DNA double-strand breaks. Carcinogenesis.

[48] Allard P, Colaiacovo MP (2010). Bisphenol A impairs the double-strand break repair machinery in the germline and causes chromosome abnormalities. Proceedings of the National Academy of Sciences of the United States of America.

[49] Inagaki A, Schoenmakers S, Baarends WM (2010). DNA double strand break repair, chromosome synapsis and transcriptional silencing in meiosis. Epigenetics.

[50] Ferguson KA, Leung S, Jiang D, Ma S (2009). Distribution of MLH1 foci and inter-focal distances in spermatocytes of infertile men. Hum Reprod.

[51] Ren H (2016). Altered Crossover Distribution and Frequency in Spermatocytes of Infertile Men with Azoospermia. PloS one.

[52] Koehler KE, Cherry JP, Lynn A, Hunt PA, Hassold TJ (2002). Genetic control of mammalian meiotic recombination. I. Variation in exchange frequencies among males from inbred mouse strains. Genetics.

[53] Liu EY (2014). High-resolution sex-specific linkage maps of the mouse reveal polarized distribution of crossovers in male germline. Genetics.

[54] Abarikwu SO, Adesiyan AC, Oyeloja TO, Oyeyemi MO, Farombi EO (2010). Changes in sperm characteristics and induction of oxidative stress in the testis and epididymis of experimental rats by a herbicide, atrazine. Archives of environmental contamination and toxicology.

[55] Radak Z, Boldogh I (2010). 8-Oxo-7,8-dihydroguanine: links to gene expression, aging, and defense against oxidative stress. Free radical biology & medicine.

[56] Zastawny TH (1998). Comparison of oxidative base damage in mitochondrial and nuclear DNA. Free radical biology & medicine.

[57] Thorslund T, Sunesen M, Bohr VA, Stevnsner T (2002). Repair of 8-oxoG is slower in endogenous nuclear genes than in mitochondrial DNA and is without strand bias. DNA repair.

[58] Hase Y (2008). Atrazine binds to F1F0-ATP synthase and inhibits mitochondrial function in sperm. Biochemical and biophysical research communications.

[59] Pepling ME (2012). Follicular assembly: mechanisms of action. Reproduction.

[60] Pepling ME, Spradling AC (2001). Mouse ovarian germ cell cysts undergo programmed breakdown to form primordial follicles. Developmental biology.

[61] Hannon PR, Niermann S, Flaws JA (2016). Acute Exposure to Di(2-Ethylhexyl) Phthalate in Adulthood Causes Adverse Reproductive Outcomes Later in Life and Accelerates Reproductive Aging in Female Mice. Toxicol Sci.

[62] Gannon AM, Stampfli MR, Foster WG (2012). Cigarette smoke exposure leads to follicle loss via an alternative ovarian cell death pathway in a mouse model. Toxicol Sci.

[63] Jefferson W, Newbold R, Padilla-Banks E, Pepling M (2006). Neonatal genistein treatment alters ovarian differentiation in the mouse: inhibition of oocyte nest breakdown and increased oocyte survival. Biology of reproduction.

[64] Gaytan F, Morales C, Manfredi-Lozano M, Tena-Sempere M (2014). Generation of multi-oocyte follicles in the peripubertal rat ovary: link to the invasive capacity of granulosa cells?. Fertility and sterility.

[65] Dutta S, Burks DM, Pepling ME (2016). Arrest at the diplotene stage of meiotic prophase I is delayed by progesterone but is not required for primordial follicle formation in mice. Reproductive biology and endocrinology: RB&E.

[66] Toyoda S (2009). Sohlh2 affects differentiation of KIT positive oocytes and spermatogonia. Developmental biology.

[67] Linnekin D (1999). Early signaling pathways activated by c-Kit in hematopoietic cells. The international journal of biochemistry & cell biology.

[68] John GB, Shidler MJ, Besmer P, Castrillon DH (2009). Kit signaling via PI3K promotes ovarian follicle maturation but is dispensable for primordial follicle activation. Developmental biology.

[69] Myers M, Middlebrook BS, Matzuk MM, Pangas SA (2009). Loss of inhibin alpha uncouples oocyte-granulosa cell dynamics and disrupts postnatal folliculogenesis. Developmental biology.

[70] Kipp JL, Kilen SM, Bristol-Gould S, Woodruff TK, Mayo KE (2007). Neonatal exposure to estrogens suppresses activin expression and signaling in the mouse ovary. Endocrinology.

[71] Kipp JL, Kilen SM, Woodruff TK, Mayo KE (2007). Activin regulates estrogen receptor gene expression in the mouse ovary. The Journal of biological chemistry.

[72] Pangas SA, Li X, Robertson EJ, Matzuk MM (2006). Premature luteinization and cumulus cell defects in ovarian-specific Smad4 knockout mice. Molecular endocrinology.

[73] Rayner JL, Wood C, Fenton SE (2004). Exposure parameters necessary for delayed puberty and mammary gland development in Long-Evans rats exposed in utero to atrazine. Toxicology and applied pharmacology.

[74] Enoch RR (2007). Mammary gland development as a sensitive end point after acute prenatal exposure to an atrazine metabolite mixture in female Long-Evans rats. Environmental health perspectives.

