# Supplementary material for: Embryonic exposure to the widely-used herbicide atrazine disrupts meiosis and normal follicle formation in female mice
Source: Sci Rep. 2017 Jun 14;7:3526. doi: 10.1038/s41598-017-03738-1 (PMC5471253; doi:10.1038/s41598-017-03738-1)
Supplement: Supplementary file 1 — Supplemental figures [file 41598_2017_3738_MOESM1_ESM.doc]

**Embryonic exposure to the widely-used herbicide atrazine disrupts meiosis and normal follicle formation in female mice**

**Aurore Gely-Pernot^1,2,3^, Souhila Saci^1^, Pierre-Yves Kernanec^1^, Chunxiang Hao^1^, Frank Giton^3^, Christine Kervarrec^1^, Sergei Tevosian^4^, Severine Mazaud-Guittot^1^ and Fatima Smagulova^1#^**

**Supplemental Data**

**
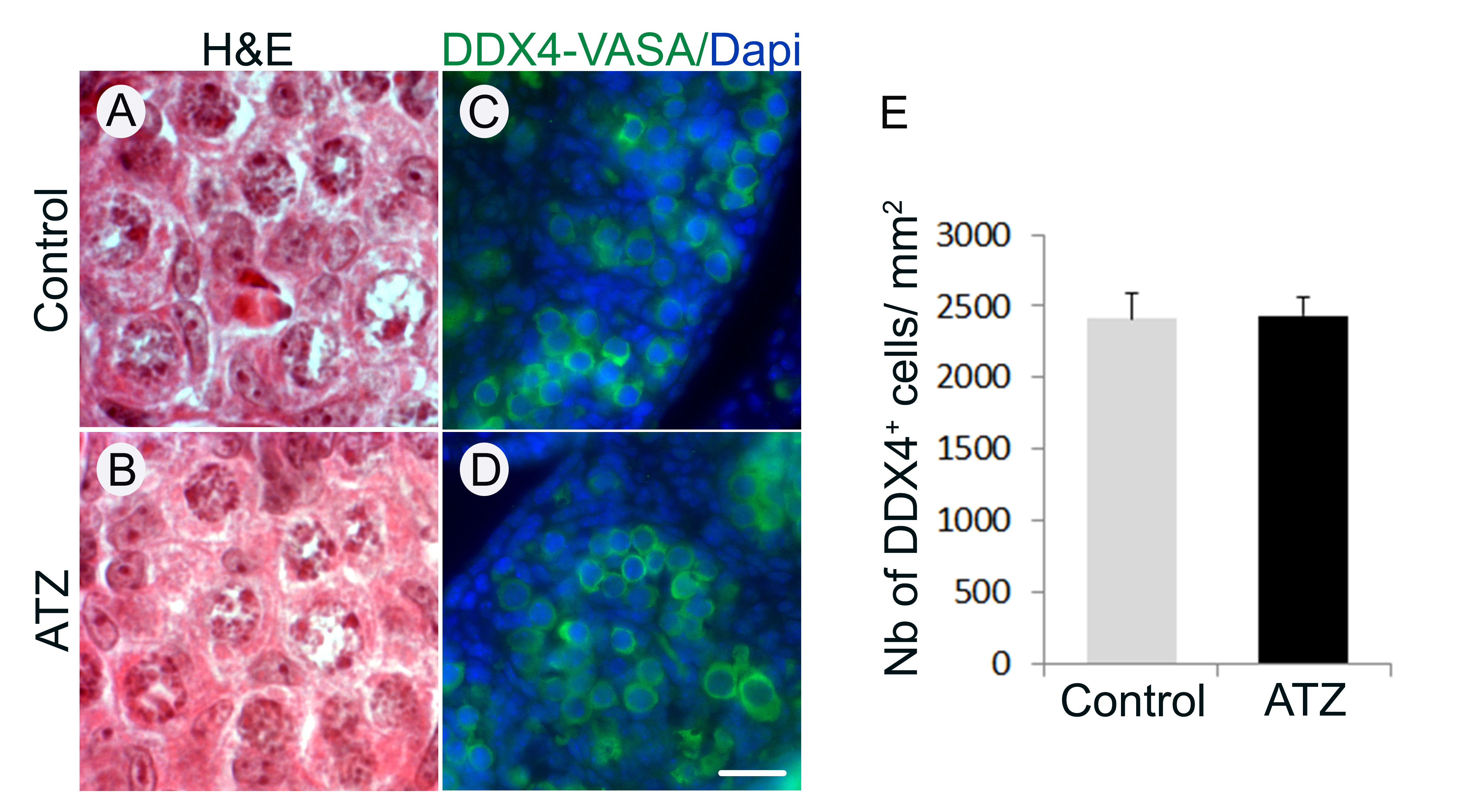
**

**Figure 1: The morphology and germ cell numbers in E18.5 ovaries are not affected by ATZ.** The histological analysis of paraffin sections (A-B) stained by hematoxylin and eosin and (C-D) immunostained with an anti-DDX4 antibody from (A-C) control and (B-D) ATZ-treated mice. (E) Quantification of the number of DDX4-positive cells per mm^2^ in control (gray bar) and ATZ-exposed (black bar) ovaries (n=6). The data are presented as the mean values +/-SEM.

**
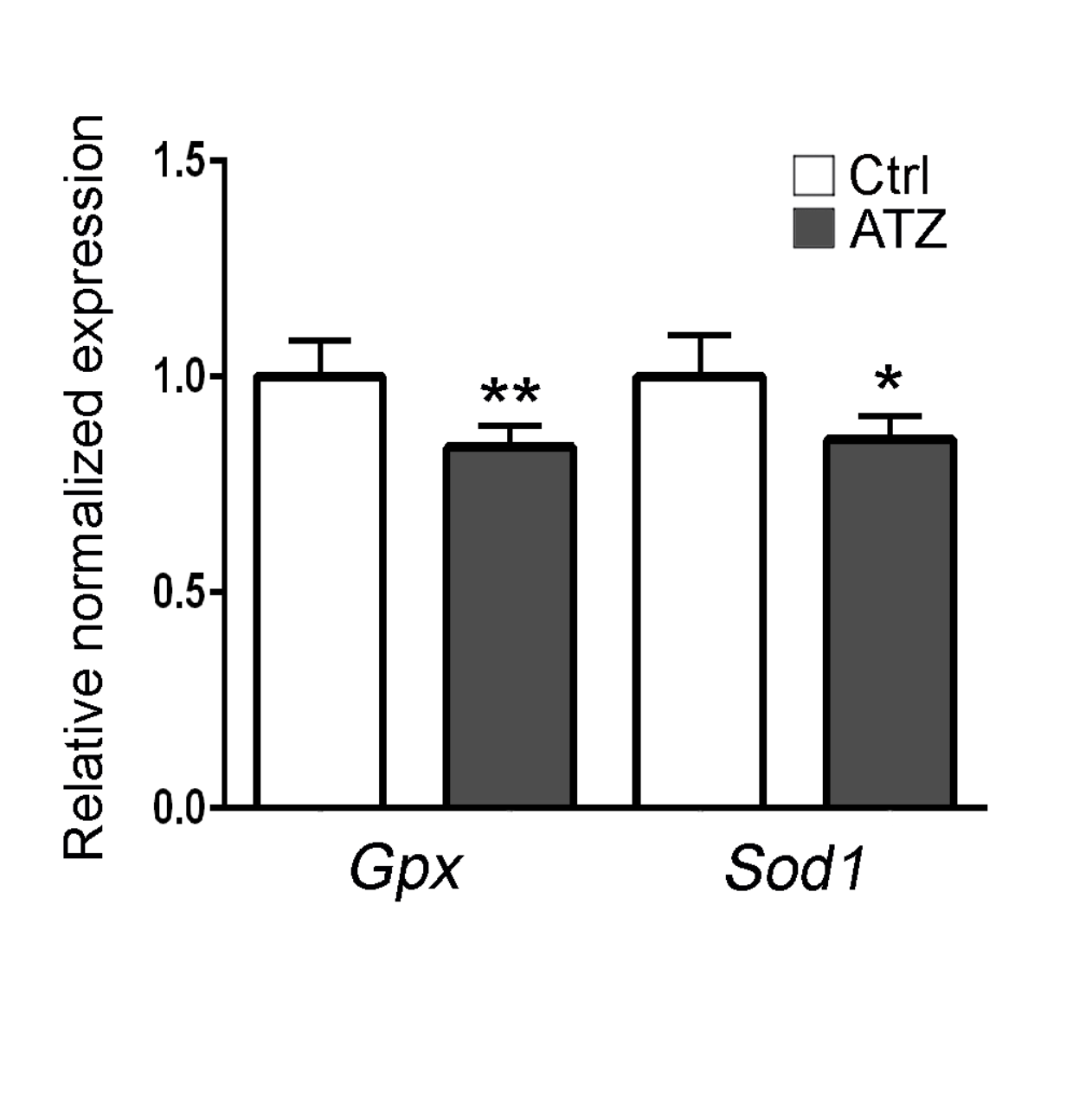
**

**Suppl. Figure 2: Embryonic exposure to ATZ affects the expression of genes involved in oxidative stress.** The expression of *Gpx1* and *Sod1* genes was analyzed by RT-qPCR using RNA from from 6-day-old ovaries from control (ctrl) and ATZ-treated animals (n=5. *p<0.05, **p <0.01, nonparametric Mann-Whitney test). The qPCR data are presented as mean values +/-SD.

**
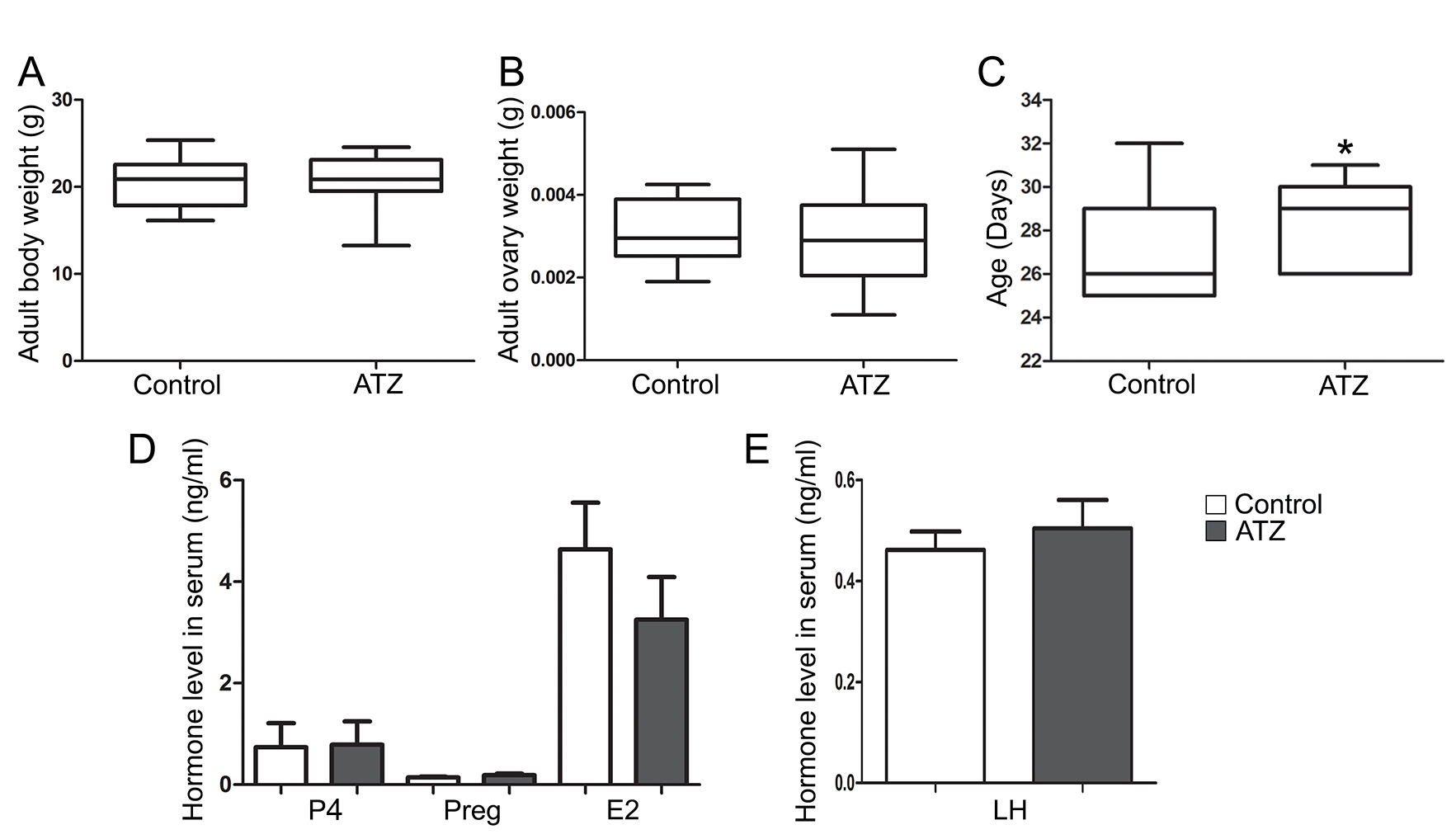
**

**Suppl. Figure 3: Exposure to ATZ does not affect the weight of ovaries or hormone levels in the serum, but delays the day of vaginal opening.** (A) The body and (B) the ovary weights of control and ATZ-treated mice on the day of vaginal opening. (C) The age of mice (days) at the time of vaginal opening, (n=15, *p<0.05, nonparametric Mann-Whitney test). (D) Progesterone (P4), Pregnenolone (Preg), Estradiol (E2) and (E) Luteinizing hormone (LH) levels in the serum, the concentration is indicated as a mean value in ng/ml +/-SD (n=15; * p≤0.05. nonparametric test)


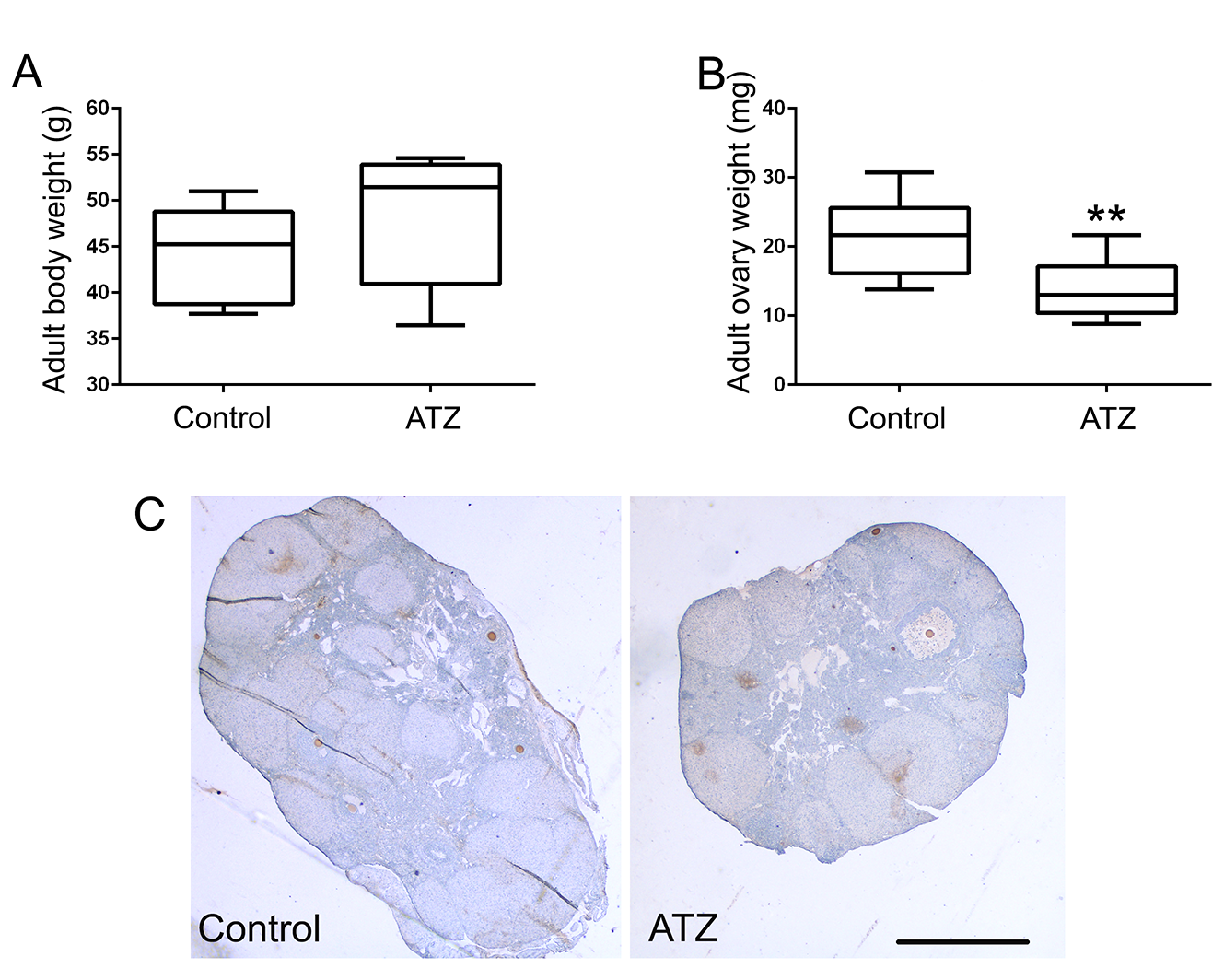


**Suppl. Figure 4: Embryonic exposure to ATZ reduces the weight of ovaries and the number of follicles in adult mice.** (A) The body and (B) the ovary weights in control (Ctrl) and ATZ- treated one- year-old mice (n=8; ** p≤0.01; nonparametric Mann-Whitney test). (C) A representative image of paraffin section of Ctrl and ATZ-treated ovary immunostained with antibody against MSY2, a specific marker of oocytes
